# Assessing NaV1.7 during tonic firing in pig C-nociceptors

**DOI:** 10.1371/journal.pone.0335081

**Published:** 2025-12-03

**Authors:** Sabrina Soares, Martin Schmelz, Richard Carr, Kyra Sohns, Roman Rukwied

**Affiliations:** Department of Experimental Pain Research, Medical Faculty Mannheim, Mannheim Center for Translational Neuroscience, University of Heidelberg, Heidelberg, Germany; University Hospital Wurzburg, GERMANY

## Abstract

Assuming the voltage-gated sodium channel (VGSC) NaV1.7 facilitates action potential generation upon slow electrical depolarization, we investigated protoxin II and TTX to target VGSC sub-types and to assess their role in C-fiber excitability when stimulated with sinusoidal single 1 Hz pulse (500 ms) and repetitive 4 Hz stimuli. We performed ex vivo extracellular compound potentials (CAP) recordings of pig saphenous nerve and in vivo pig single nerve fiber (SNF) recordings of heat- mechanosensitive (“polymodal”) nociceptors (C-HT) and low- threshold mechanoresponsive C-fibers (C-LTMR) upon electrical 1 and 4 Hz sinusoidal stimulation, which evoke a discharge burst and a tonic response, respectively. Both toxins reduced C-CAP amplitudes and conduction velocity. Number of action potentials evoked by low-intensity phasic (1 Hz) or tonic (4 Hz) sinusoidal stimulation were reduced in C-HT nociceptors after protoxin. In C-LTMR fibers, protoxin reduced the number of action potentials to 4 Hz, but did not affect 1 Hz discharges. The toxins did not increase the delay of action potential initiation of C-CAPs or during SNF. Our results confirm the functional role of NaV1.7 to tonic supra-threshold electrical 4 Hz sinusoidal action potential firing in C-fibers. Protoxin reduced AP discharges to low-intensity phasic 1 Hz stimuli in C-HT nociceptors but not C-LTMR touch fibers. This finding suggests a differential functional role of NaV1.7 between C-fiber classes. Peripheral NaV1.7 blockade seems to increase the depolarization level required for C-nociceptor activation, and this might be relevant to target clinically ongoing pain.

## Introduction

The discovery of gain-of-function mutations of sodium channel NaV1.7 leading to inherited erythromelalgia [1] provided the molecular basis of clinically relevant neuropathic pain. Mechanistically, increased nociceptor excitability was linked to hyperpolarized activation thresholds of mutated NaV1.7 channels [2] and to increased discharge rates in response to supra-threshold depolarizing stimuli [3]. Findings on NaV1.7 voltage dependency are based on patch clamp recordings of cultured neurons but cannot be performed in nociceptive endings *in vivo*. To improve translation, we used extracellular electrical C-nociceptor stimulation protocols that can be correspondingly used in humans. Stimuli comprised slow depolarizing currents (sinusoidal waveform at 4 Hz) to provoke single action potentials in C-nociceptors [4]. Even slower depolarization (1 Hz sine wave 500 ms) evokes burst discharge in polymodal nociceptors [5] mimicking tonic current injections under patch clamp conditions that initiate trains of action potentials [3]. Using both sinusoidal stimulation protocols, we explored protoxin and TTX for voltage-gated sodium channel (VGSC) blockade and their effects on specific C-fiber class excitability. Pig single nerve fiber recordings (SNF) ensured that C-fiber classes correspond to their human counterparts [6].

The voltage-gated sodium channel NaV1.7 in nociceptors has been characterized as a threshold channel that can amplify the generator potential by ramp currents [7]. NaV1.7 aids to set activation thresholds [8] and ensures axonal conduction of unmyelinated nociceptors [9]. Based on its slow repriming kinetics, NaV1.7 might be less important for high frequency discharge [8], but it was shown recently that it also contributes to repetitive firing, even though with a smaller action potential current [10]. We therefore expected blockade of NaV1.7 to increase activation thresholds and reduce axonal conduction of C-CAPs and particularly in single polymodal C-nociceptors. We aimed to clarify to which extent NaV1.7 blockade would impair acute C-fiber bursting discharge upon prolonged 1 Hz 500 ms depolarization or reduce tonic action potential firing upon repetitive 4 Hz sinusoidal stimulation. Thereby, our results could provide mechanistic insights to neuropathic pain pathology.

## Materials and methods

### Animals

Experiments followed the guidelines for animals’ welfare according the Federal Republic of Germany and approved by the regional governmental ethics committee (Karlsruhe, Germany, approval number G-78/18). Domestic German Landrace pigs (*Sus Scrofa domesticus*, 20–25 kg, age 12 ± 4 weeks) were sedated with Dormicum® (1 mg/kg, Roche, Switzerland) and Stresnil® (5 mg/kg, Jannsen Pharmaceutica, Belgium). General anesthesia was maintained by intravenous Narcoren® infusion (15–20 mg/kg/h, Rhone Merieux, Germany). Animals were volume-controlled ventilated (5–10 ml/kg TV) and monitored for EtCO_2_, ECG, temperature, SPO_2_ and NIBP (Primus® Dräger Germany and uMEC12 Vet®, Mindray China). Euthanasia was performed with an extra bolus Narcoren® (30 mg/kg) followed by supersaturated potassium chloride (1 ml/kg, Sigma-Aldrich, Germany) administered in the jugular vein.

### Compound action potential (CAP) recordings ex vivo

#### Nerve preparation and CAP recordings.

*Ex vivo* pig saphenous nerve segments were de-sheathed and cut into segments of approximately 10–12 mm in length. Individual nerve fascicles were placed in a custom-made organ bath (volume 750 µl) perfused with HEPES buffer. On either side of the bath, nerves were drawn through glass pipettes and sealed with thin silicone layers. The distance between the HEPES buffer filled glass pipettes varied between 4–7 mm. Electrical currents (0–50 µA) were delivered as 1 ms rectangular or 125 ms sinusoidal pulses (constant current stimulator A395, WPI, FL, USA) across the nerve via two silver wires, one placed inside the glass pipette (anode) and the other in the organ bath (cathode). At the other glass pipette, a second pair of silver wires served as recording electrodes.

### Single nerve fiber (SNF) recordings in vivo

#### Pig nerve preparation.

The saphenous nerve was exposed, dissected from connective tissue, the subcutaneous tissue separated and the skin flap sutured to a metal ring to form a pool filled with paraffin oil. Nerve fascicles were de-sheathed and teased fibers processed for single-unit recording [6,11].

#### SNF recordings.

Receptive fields of sensory afferents were mechanically localized and rectangular supra-threshold electrical stimuli (20 mA, 0.5 ms pulses) delivered at 0.25 Hz via non-insulated microneurography needles until time-locked action potential were induced. Needles were inserted intradermally at this site. Pre-amplified signals (INV Biosignal Amplifier, Avere Solutions UG, Erlangen, Germany) were amplified, filtered (bandwidth 100–3000 Hz, Model 3364, Krohn-Hite Corp., Brockton, USA), audio monitored and displayed (DAPSYS 8.0 software package, ©Brian Turnquist, Minnesota, US). Electrical sine wave current profiles of 1 Hz and 500 ms duration (intensities ranging from 0.02–10 mA) and 4 Hz (250 ms/cycle) of 0.05–0.8 mA amplitudes were controlled by DAPSYS 8.0 and applied within the receptive field using a constant current stimulator (A395, WPI, US) via a pair of transcutaneous L-shaped platinum-iridium electrodes, (diameter 0.4 mm, distance 2 mm). Single 1 Hz sine wave bouts were applied with increasing intensities from 0.02 to 10 mA in 10 s intervals delivered in three stimulation ranges of 0.02–0.1 (interval rise 0.02 mA), 0.2–1 (interval rise 0.2 mA), 2–10 (interval rise 2 mA). The 1 Hz evoked number of action potentials were grouped and analysed according stimulation range. The 4 Hz sine wave stimulus was delivered continuously for 1 minute (240 pulses) at each stimulus intensity of 0.05, 0.1, 0.2, 0.4, 0.8 and 1.2 mA.

#### Nerve fiber classification.

Single C- fibers were classified according to their conduction velocity (CV) and responsiveness to mechanical, heat and electrical stimulation online and verified offline. In brief, high-threshold mechano-thermal-sensitive C-nociceptors (“polymodal” nociceptors, C-HT) can follow 100 Hz supra-threshold rectangular electrical stimulation [12], respond to mechanical force evoked by Semmes-Weinstein monofilaments [13] and discharge to CO2-Laser 45–50°C stimulation (SIFEC, Ferrieres, Belgium). Low-threshold mechano-sensitive C-fibers (“C-touch fibers”, C-LTMR) are sensitive to soft brush and are not activated by CO2-Laser heat.

### Chemicals and solutions

#### Extracellular physiological solution (HEPES buffer solution).

Composition (in mM): NaCl 118; KCl 3.2; HEPES 6; Na^+^ gluconate 20; CaCl_2_ 1.5; MgCl_2_ 1.0; D-Glucose 5.55; adjusted to pH 7.4. Glucose, MgCl_2_ and CaCl_2_ were added to the solution prior to each experiment.

#### Phosphate-Buffered Saline (PBS).

PBS was used to constitute tetrodotoxin (TTX) citrate, µ-conotoxin (PIIIa) and protoxin II (ProTx II). Composition (in mM) NaCl: 1037; KCl: 27; Na_2_HPO_4_: 100; KH_2_PO_4_: 18; 10-fold dilution in distilled water before use.

#### Na+ channel blockers.

Tetrodotoxin citrate (TTX) (T-550), µ-conotoxin PIIIa (STC-400), and protoxin II (STP-100) were purchased from ALOMONE labs® (Jerusalem, Israel), lidocaine hydrochloride monohydrate from Sigma Aldrich (Munich, Germany). Stock solutions were prepared in PBS and diluted in HEPES on the experimental day. TTX was prepared at 10nM, 100nM and 1µM concentrations and volumes of 100 µl were injected intradermally into the receptive field of the unit for SNF recordings. A four-parameter logistic (4PL) model was used to fit the number of APs in response to sine 1 Hz to a sigmoidal dose-response curve for C-HT (supplement S1A Fig) and C-LTMR (supplement S1B Fig). In another series of SNF recordings, responses to electrical stimulation were recorded before and after injections of 100µl protoxin 2.5µM and TTX 1µM into the receptive fields.

For CAP recordings, concentration-response curves were performed from 0.1nM to 10µM for protoxin-II and 1pM to 10µM for TTX. Triplicates of CAP maximum amplitudes (µV) were collected for each concentration and sigmoidal fitted. TTX at 1µM and lidocaine 10mM were perfused into the recording bath. Protoxin 5µM and µ-conotoxin PIIIA 20µM diluted in 1 ml of HEPES were added directly into the recording bath. Protoxin II and µ-conotoxin PIIIa were used to target NaV1.7 [14] and NaV1.6 [15], respectively. Concentrations for the *in vivo* and *ex vivo* experiments were chosen based on the concentration-response curves.

### Recording parameters

#### CAP recordings.

CAP data were analyzed in Igor Pro 7 (WaveMetrics, Lake Oswego, OR, US). Maximum CAP-amplitudes (µV) were recorded from peak to peak and latencies (ms) to rectangular stimuli (L_R_) measured as the time between electrical stimulus and half-maximum rising peak of a C-fiber compound potential (C-CAP) in response to both supra-maximum and threshold intensities. Conduction velocity (m/s) was calculated by dividing conduction distance (between stimulation and recording electrode) by L_R_. The latency of sine wave C-CAPs at supra-maximum and threshold intensities was corrected by subtraction of L_R_ (“initiation delay”), thereby calculating the actual time required to initiate the action potential by sine waves. All parameters were compared within the same nerve fascicle before and after sodium channel blockers and normalized to baseline by division.

#### SNF recordings.

Stimulus thresholds and latencies to rectangular stimuli (L_R_) were transferred to Excel 2019, and action potential data to slow depolarising stimuli analyzed using DAPSYS 8.0. Responses to 1 Hz bouts were measured as number of action potentials per stimulus intensity. For sine 4 Hz stimuli the total number of action potentials per stimulus intensity delivered for 1 minute was divided by 240 pulses. Conduction distance (mm) was measured between the recording electrode and the needles used for stimulation. The latency (ms) was the time from electrical rectangular stimulation onset (at supra-threshold 20 mA) to an action potential signal at the recording electrode. Conduction velocity (m/s) was calculated by dividing conduction distance by latency. Latency (ms) for sine wave 1 Hz (500 ms bouts) was defined as the time to the first AP. The stimulus intensity at which at least 3 APs were evoked was defined as stimulus threshold. The AP latency upon sine wave 4 Hz pulses was converted to phase (π) of the sine wave at which the first AP occurred during the 1-minute stimulation at each stimulus intensity tested. Sine wave 1 and 4 Hz latencies were corrected by subtracting L_R_.

#### Statistics.

Data was tested for normality (Shapiro–Wilk test) and analyzed in GraphPad Prism (GraphPad 10.1.0, Boston, Massachusetts USA). The effect of the toxins on C-CAP amplitudes, latencies and conduction velocity were normalized to baseline by division and analyzed with repeated measures One-way ANOVA followed by Tukey post-hoc test. C-CAP initiation delay compared at the same stimulus intensity before and after toxin administration was analysed using Kruskal-Wallis test followed by Dunn’s post-hoc test. For SNF recordings, Two-way ANOVA with Tukey post-hoc test was used to compare the effect of stimulus intensities (factorial group “intensity”) and toxin treatments (factorial group “treatment”) as well as the interaction between the two variables (intensity x treatment) on number of action potentials and conduction velocity for C-HT and C-LTMR. The time and phase of first action potential (sine 1 Hz) for both C-HT and C-LTMR was tested using Kruskal-Wallis test followed by Dunn’s post-hoc test when applicable. Grouped data are presented as mean and standard error (SEM). Levels of significance are indicated as *^/#^*p < 0.05*, **^/##^*p < 0.01*, ***^/###^*p < 0.001* or ****^/####^*p < 0.0001*.

## Results and discussion

### C-compound action potential (C-CAP) amplitudes and conduction velocity (CV) were reduced by protoxin II

**Fig 1:**
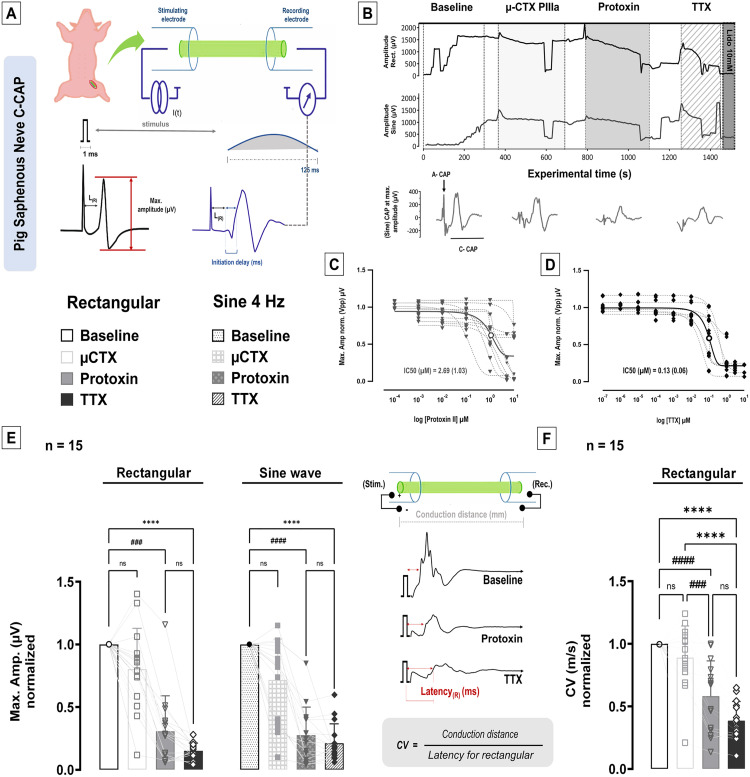
Protoxin reduces maximum amplitude and CV in ex vivo C-CAP. **(A)** Schematic representation of ex vivo compound action potential (CAP) recordings of pig saphenous nerve, depicting the measurements of maximum amplitude, latency for rectangular electrical stimulus L_R_, and initiation delay for sine wave stimulus. Note that initiation delay in response to the sinusoidal stimulus is corrected to L_R_. (Top panel, **B)** A complete compound potential recording is displayed, in which maximum amplitudes were measured for rectangular and sine wave stimulations and (lower panel, **B)** C-CAP potential signals were exemplified after the addition of µ-CTX PIIIa 20µM (and the corresponding blockade of the A-CAP, highlighted by the black arrow), protoxin 5µM, tetrodotoxin (TTX) 1µM, and finally lidocaine 10mM delivered for unspecific Na + -channel blockade. **(C)** The IC50 of protoxin was 2.69 (±1.03) (n = 11, in µM). **(D)** TTX IC50 was 0.13 (±0.06) (n = 7, in µM). **(E)** There was no effect of µ-conotoxin in C-CAP amplitudes (One-way ANOVA, rect. p = 0.33, sine p = 0.07). To baseline condition normalized C-CAP amplitudes were reduced after protoxin and TTX (n = 15, One-way ANOVA, Tukey post-hoc test, rect. and sine p < 0,0001). There was no difference in C-CAP amplitudes between protoxin and TTX treatment (rect. p = 0.34, sine p = 0.89). **(F)** Specimen of a C-CAP recording and corresponding CV calculation (left panel). The conduction velocities (CV) of C-CAPs (right panel) were reduced after protoxin and TTX, but no effect was seen after µ-CTX (One-way ANOVA, Tukey post-hoc test, **p < 0.01, ***p < 0.001, ****p < 0.0001). C-CAP amplitude and CV are normalized to values recorded prior to toxin administration (baseline) and presented as mean (SEM). Levels of significance are indicated as ****p < 0.0001 for TTX and ### p < 0.001 or ####p < 0.0001 for protoxin.

C-fiber axons in isolated segments of pig saphenous nerve were excited by conventional 1 ms rectangular electrical pulses and a slow depolarization (4 Hz sinusoidal; Fig 1A), both yielding maximal C-CAP response (Fig 1B, baseline). Sodium channel blockers were applied to 15 C-CAP recordings in a fixed sequency, beginning with µ-conotoxin PIIIa (20µM), followed by protoxin II (5µM), then tetrodotoxin (TTX, 1µM), and ultimately lidocaine (10mM) (Fig 1B, top panel). Blocking NaV1.6 with µ-conotoxin PIIIa abolished the A-fiber CAP component (black arrow, lower panel, Fig 1B).

The IC50 in µM for protoxin II was 2.69 (±1.03) (n = 11, Fig 1C) and 0.13 (±0.06) µM for TTX (n = 7, Fig 1D). µ-conotoxin PIIIa did not reduce the C-CAP amplitude to rectangular or sine wave stimuli (One-way ANOVA, rect. *p = 0.33*, sine *p = 0.07*) despite evidence for Nav1.6 in some unmyelinated axons [16]. In contrast, protoxin resulted in a significant reduction in C-CAP amplitude (One-way ANOVA, Tukey post-hoc test, rect. and sine *p < 0.0001*). Adding TTX 1µM also reduced the C-CAP amplitudes (rect. and sine *p < 0.0001*), but without further reduction compared to protoxin (rect. *p = 0.34*, sine *p = 0.89*) (Fig 1E). Maximum C-CAP amplitudes (µV) normalized to baseline in response to rectangular current stimuli were initially 2035 (±353.3), fell to 1663 (±329.9) after µ-conotoxin, further to 570.7 (±127.5) after protoxin and finally 294.8 (±61.45) after TTX. Sine wave induced maximum C-CAP amplitudes in the absence of sodium channel blockers was 1597 (±301.5) µV. This C-CAP response to sine wave stimulation was also reduced by µ-conotoxin to 1229 (±314.7), to 327.9 (±58.32) after protoxin and to 285 (±57.31) after TTX. Notably, there was a significant difference between rectangular and sine wave induced C-CAP amplitudes before (*p = 0.03*) but not after µ-conotoxin, protoxin and TTX administration. C-CAP signals were completely blocked by lidocaine 10mM (positive control).

As previously mentioned, conduction velocity (CV, m/s) was calculated by dividing conduction distance by L_R_ (Fig 1F, left panel). Both protoxin II and TTX reduced normalized CAP conduction velocity significantly (One-way ANOVA, Tukey post-hoc test, *p < 0.001* and *p < 0.0001*), whereas µ-conotoxin PIIIA had no effect (*p = 0.36,* Fig 1F, right panel). TTX further reduced CV after protoxin. Although this reduction was non-significant (*p = 0.051*), it may indicate the impact of TTX sensitive NaV channels others than sub-types 1.6 and 1.7 on neuronal conductance. CVs of C-CAPs were 0.27 (±0.03) before, 0.23 (±0.02) after µ-conotoxin, 0.15 (±0.02) after protoxin and 0.1 (±0.01) after TTX.

The C-CAP amplitude is an excellent measure of the number of electrically activated fibers and synchronized action potentials, but does not differentiate specific C-fiber sub-types which requires single fiber recordings.

### Differential effect of protoxin II in single C-HT and C-LTMR

**Fig 2:**
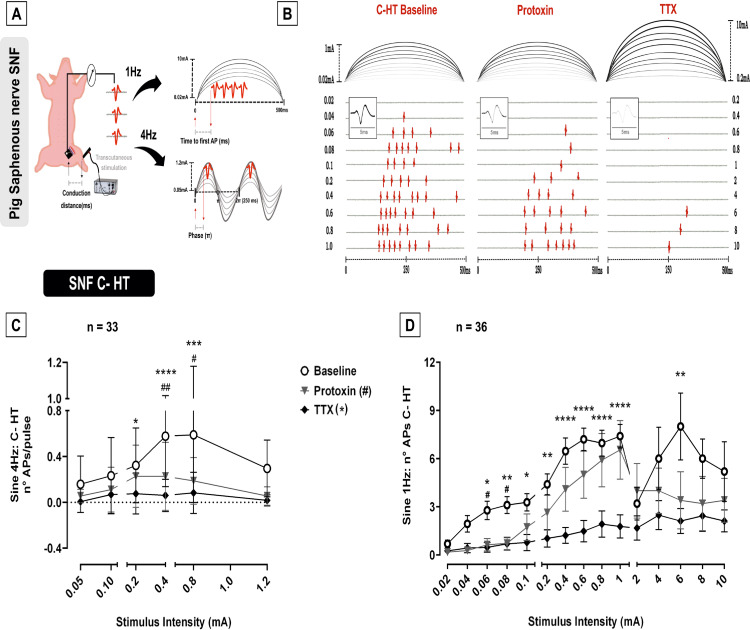
C-HT single units reduced response to sine wave 1 and 4 Hz stimulation after protoxin. **(A)** Schematic representation of SNF recordings of pig saphenous nerve, depicting the conduction distance (left panel), the time of the first action potential (AP) for sine wave 1 Hz stimulation (right panel, top) and the phase at first AP for sine 4 Hz before correction to latency for rectangular electrical stimulus (right panel, bottom). The bursts of action potentials in response to sine 1 Hz and the single AP per sine wave 4 Hz cycle is also schematically represented. **(B)** Specimen of C-HT electrically stimulated using sine 1 Hz, in which the number of APs are displayed in response to increasing stimulus intensities (top – bottom) varying from 0.02–10 mA at baseline before toxin administration (left) and after protoxin (middle) and TTX (right). The occurrence time of APs in relation to the 500 ms stimulus are shown for the unit and an overlay of the responses depicting the AP shape (inlet). **(C)** Protoxin reduced the number of APs per pulse (±SEM) of polymodal nociceptors for sine 4 Hz (Two-way ANOVA, Tukey post-hoc test, p < 0.0001). There was no significant difference between protoxin and TTX in the number of APs (p > 0.05). **(D)** The injection of both protoxin and TTX reduced the number of APs (±SEM) of polymodal nociceptors to sine 1 Hz stimulus, particularly at low stimulus intensities (Two-way ANOVA, Tukey post-hoc test, p < 0.0001). There was no significant difference between protoxin and TTX blockade in the number of APs (p > 0.05). C-HT nociceptors respond in a stimulus-dependant manner to both sine 4 Hz (One-way ANOVA, p = 0.0046) and 1 Hz (One-way ANOVA, p < 0.0001). Levels of significance are indicated as *p < 0.05, **p < 0.01, ***p < 0.001 or ****p < 0.0001 for TTX and #p < 0.05 or ##p < 0.01 for protoxin.

The difference in discharge pattern upon 4 and 1 Hz sine wave stimuli during SNF recording is schematically represented in Fig 2A. “Number of APs” and “phase” of the first AP (1 sinusoidal cycle = 360° = 2π) were analyzed for the 4 Hz stimulation; “Number of APs” and “time to first AP” (corrected for rectangular stimulation latency, L_R_) were determined for the 1 Hz stimulation. Threshold currents were determined for both stimulus paradigms and all parameters compared before and after protoxin and TTX injection into the receptive field. The concentration of 50% inhibition (IC50) was 0.58 (±0.15) µM of TTX for C-HT (n = 9, S1A Fig) and 0.83 (±0.22) µM TTX for C-LTMRs (n = 8, S1B Fig).

36 C-HT (“polymodal”) nociceptors were recorded during sine wave 1 Hz (500 ms) pulse (specimen Fig 2B) and 33 units during sine wave 4 Hz stimulation delivered for 1 minute (data supplement S1 Table). Protoxin and TTX reduced the number of APs to sine 4 Hz (Fig 2C, Two-way ANOVA, *p < 0.0001*) and to 1 Hz, particularly at 0.06 and 0.08 mA (Fig 2D, Two-way ANOVA, *p < 0.0001*). In addition, excitation thresholds for sine 1 Hz increased in C-HT (supplement S2 Table) after TTX (One-way ANOVA, *p = 0.0003*) but not after protoxin (One-way ANOVA, *p = 0.13*). Polymodal nociceptors responded intensity-dependently to single 1 Hz pulses (One-way ANOVA, *p < 0.0001*) and sine wave 4 Hz (One-way ANOVA, *p = 0.0046*). There was no interaction between “treatment” and “intensity” for sine 4 Hz (Two-way ANOVA, *p = 0.136*). In contrast, a treatment-intensity interaction was observed for 1 Hz sine wave stimulation (Two-way ANOVA, *p = 0.0095*).

**Fig 3:**
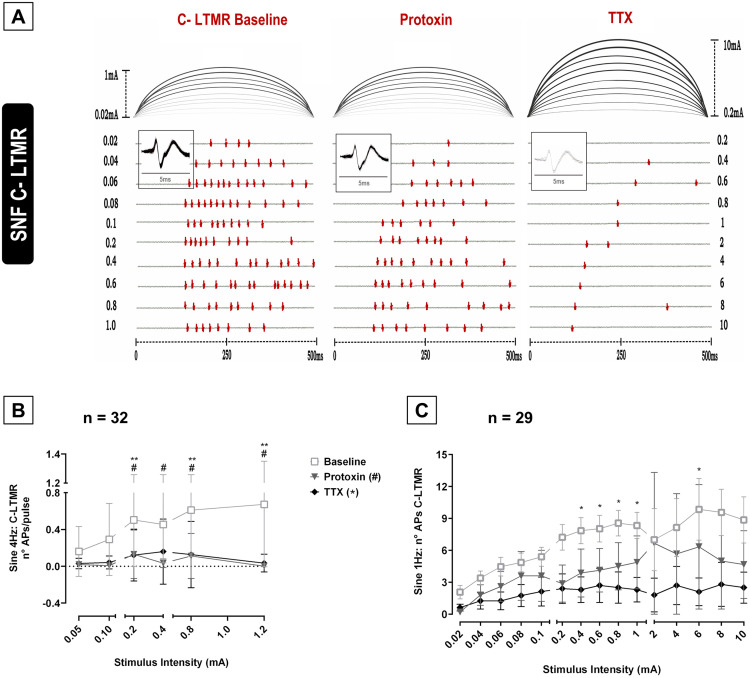
C-LTMR responses in vivo single nerve fiber (SNF) recordings to sine wave 1 and 4 Hz stimulation. **(A)** Specimen of a C-fiber with low mechanical threshold (C-LTMR), electrically stimulated with sine 1 Hz before toxin administration (baseline, left panel) and after intradermal protoxin (ProTx, middle) and TTX (right panel) injections (100 µl) into the receptive field of the unit. The number of action potentials (APs) are displayed in response to increasing stimulus intensities (top to bottom) varying from 0.02–10 mA. The time of AP occurrence is depicted for the unit and an overlay of the AP responses shows the action potential shape (inlet). **(B)** Both protoxin and TTX reduced the number of APs (±SEM) of C-LTMRs for sine 4 Hz when compared to baseline condition (Two-way ANOVA, Tukey post-hoc test, p < 0.0001). **(C)** The injection of TTX reduced the number of AP of C-LTMR fibers for sine 1 Hz stimulus p < 0.0005) while no effect of protoxin was observed in reducing bursts of action potentials (p > 0.05). Note that C-LTMR fibers responded in a stimulus-dependant manner to sine 1 Hz (p = 0.049) but not to sine 4 Hz stimulation (p = 0.11). Levels of significance are indicated as *p < 0.05 or **p < 0.01 for TTX and #p < 0.05 for protoxin.

C-LMTR fibers were stimulated with 4 Hz sine waves for 1 minute (240 pulses, n = 32) and with sine wave 1 Hz (500 ms bouts, n = 29, summary of results see supplement S1 Table). C-LTMR fibers responded to sine wave 1 Hz stimulation in an intensity-dependent manner (Specimen Fig 3A, One-way ANOVA**,** *p = 0.049*). The intensity-response relationship to sine wave 4 Hz stimulation seen in C-HT nociceptors was not apparent in C-LTMRs (Fig 3B, ANOVA, *p* = *0.11)*. Injection of both protoxin and TTX into the receptive fields, reduced the response in C-LTMR to sine wave 4 Hz (Fig 3B, Two-way ANOVA, Tukey post-hoc test, *p < 0.0001*). TTX increased excitation thresholds for sine 1 Hz in C-HT nociceptors (One-way ANOVA, *p < 0.001*), but not significantly in C-LTMR fibers (*p = 0.58*, supplement S2 Table). TTX reduced 1 Hz induced AP bursts in C-LTMRs significantly (Fig 3C, Two-way ANOVA, Tukey post-hoc test *p < 0.0005*), while protoxin did not reduce AP numbers for any 1 Hz intensity range tested (*p* > *0.05)*. Similar to C-HT, there was no significant difference among protoxin and TTX in the number of APs to sinusoidal 4 Hz or 1 Hz stimuli (*p* > *0.05*), and there was no interaction between “intensity” and “treatment” for both stimuli.

### Time to first action potential upon depolarization

**Fig 4:**
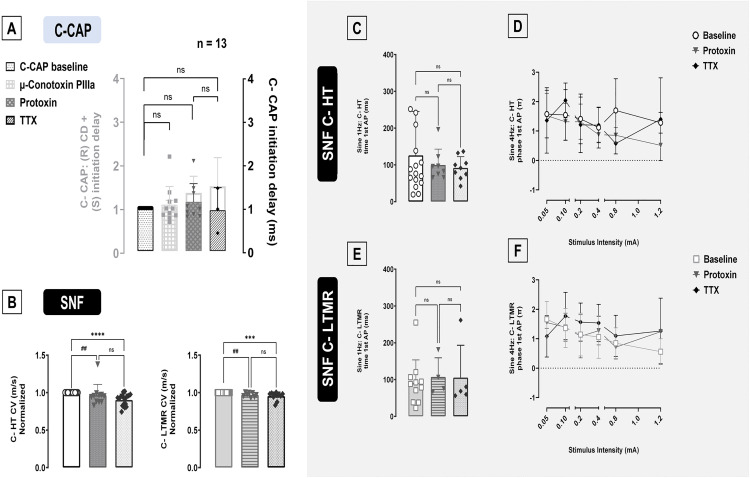
Initiation latencies in ex vivo (C-CAP) and in vivo (SNF) recordings. **(A)** The latency (light grey columns and left y-axis) or initiation delay (dark grey columns and right y-axis, after correction for L_R_ or conduction delay) at threshold for sine wave 4 Hz stimulus was not affected by both protoxin or TTX (One-way ANOVA, p = 0.29). Both protoxin and TTX reduced conduction velocities normalized to baseline for polymodal C-HT nociceptors (B panel left, n = 24, One-way ANOVA, Tukey post-hoc test, p < 0.0001) and C-LTMR units (B panel right, n = 16, p = 0.0002) in single nerve fiber recordings (SNF). The time of first AP (±SEM) in C-HT nociceptors for sine wave 1 Hz **(C)** (Kruskal-Wallis test, p = 0.97) and 4 Hz **(D)** (Two-way ANOVA, p = 0.14) stimulation was not increased by both protoxin and TTX. **(E and F)** Responses of low-threshold mechanoreceptors (C-LTMRs) to 1 Hz **(E)** (Kruskal-Wallis test, p = 0.823) and 4 Hz stimuli **(F)** (Two-way ANOVA, p = 0.23) were not altered by the injection of either protoxin or TTX into the receptive fields of the recorded fibers. Levels of significance are indicated as ***p < 0.001 or ****p < 0.0001 (represented as ##p < 0.01 for protoxin).

None of the toxins tested influenced the time required to generate CAPs at sinusoidal threshold intensities (Fig 4A, One-way ANOVA, *p = 0.29*) whereas it reduced conduction velocity (see Fig 1F).

Similarly to C-CAP, both TTX and protoxin slowed conduction velocity (CV) normalized to baseline in C-HT nociceptors (Fig 4B panel left, n = 24, One-way ANOVA, Tukey post-hoc test, p < 0.0001) and C-LTMR fibers (Fig 4B panel right, n = 16, One-way ANOVA, Tukey post-hoc test p < 0.001) but had no effect on sine 1 Hz time to first AP (Fig 4C, n = 16, Kruskal-Wallis test, p = 0.97) or sine 4 Hz phase of first AP generation in C-HT nociceptors (Fig 4D, Two-way ANOVA, p = 0.14), and similarly in C-LTMRs stimulated with 1 Hz (Fig 4E, n = 11, Kruskal-Wallis test, *p = 0.823*) or 4 Hz (Fig 4F, Two-way ANOVA, *p = 0.23*).

The average raw values of CV (m/s) for C-HT nociceptors were 0.88 (±0.04) before, 0.84 (±0.05) after protoxin and 0.82 (±0.05) after TTX. To baseline condition normalized CVs for C-HT were 0.97 (±0.04) after protoxin (One-way ANOVA, *p < 0.002*) and 0.9 (±0.02) after TTX (One-way ANOVA, *p < 0.0001*). C-LTMR’s CV were 1.18 (±0.08) before, 1.05 (±0.06) after protoxin and 1.13 (±0.11) m/s after TTX. Normalized CV for C-LTMR were 0.97 (±0.01) after protoxin (One-way ANOVA, *p < 0.01*) and 0.95 (±0.02) after TTX (One-way ANOVA, *p < 0.001*).

As higher stimulus currents could reduce C-CAP latencies (supplement S2A Fig, top right panel) we also compared the C-CAP initiation delay for the same threshold stimulus intensities before and after toxin administration. Indeed, both µ-conotoxin and TTX but not protoxin showed a significant increase in initiation delay compared to baseline C-CAP at the same stimulus intensity (supplement S2A Fig, n = 13, Kruskal-Wallis, Dunn’s post hoc test, CTX *p < 0.05*, protoxin *p > 0.05,* TTX *p < 0.002*). The values of initiation delay (ms) were 37.57 (±2.84) before and 48.17 (±3.24) after µ-conotoxin; 37.68 (±2.92) before and 47.75 (±4.15) after protoxin; 31.16 (±2.21) before and 51.92 (±5.24) after TTX.

Additionally, we analyzed the effect of the toxins on time (1 Hz) and phase (4 Hz) of first AP normalized to baseline for C-HT and C-LTMR fibers stimulated at same stimulus intensities before and after toxins were injected into the receptive fields. No effect of the toxins was seen in time (supplement S2B Fig, n = 11, Kruskal-Wallis, *p = 0.315*) or phase (supplement S2C Fig, n = 20, *p = 0.414*) of first AP generation in C-HT nociceptors. Similarly, C-LTMRs also did not show any effect of the toxins on the time of first AP when normalized to baseline condition at the same 1 Hz stimulus intensities (supplement S2D Fig, n = 8, *p = 0.089*) or the phase of first AP at the same 4 Hz stimulus intensities (supplement S2E Fig, n = 9, *p = 0.12*).

We investigated the impact of toxins targeting voltage gated sodium channels on electrical sinusoidal stimulation of porcine polymodal C-HT nociceptors and C-LTMR units that have corresponding sensory and axonal characteristics to humans [6] in comparison to mouse [17]. This species consideration is of particular importance as also expression of the voltage gated sodium channels NaV1.8 and NaV1.9 differs between human and mouse neurons [18]. In terms of NaV1.7, however, a broad similarity of the SCN9A sequence was obtained between these species [19] and can be assumed also for pigs [20]. We selected protoxin II and TTX aiming for NaV1.7 [21,22] and NaV1.1-NaV1.7 [23,24] blockade, respectively, and used concentrations based on our concentration-response curves obtained from pig nerves *in vitro*. We recorded potentially important differences in the response of C-LTMR and C-HT neurons. Protoxin reduced burst discharge of polymodal C-nociceptors induced by 1 Hz sinusoids at low current intensities while its affect on the discharge of C-LTMR fibers was less pronounced. TTX was effective in both fiber classes at any stimulus intensity. These differences between protoxin and TTX point out the role of NaV1.7 for nociceptor excitability at threshold, and differential sensitivity to NaV1.7 in non-nociceptors like C-LTMR. Alternatively, both toxins at the concentrations used may have been acting on other ion-channels, possibly suggesting that other TTX-sensitive isoforms other than NaV1.7 are involved in sinusoidal excitability of C-LTMR fibers. Transcriptomics of mouse DRG neurons identified no differential expression of NaV1.7 between C-LTMRs and C-HT neurons [25], but specific NaV1.7 expression quantification in pig neurons is not yet available. Moreover, a computational model suggests NaV1.7 to be crucial for C-HT neuronal activity [26] and some studies suggest that NaV1.7 is required for normal C-LTMR function and affective touch [27]. Indeed, protoxin and TTX reduced 4 Hz evoked AP discharge in both C-HT nociceptors and C-LTMR fibers. The continuous one-minute stimulation is expected to hyperpolarise the axon [28], thereby increasing NaV1.7 availability. Such activity-dependent increase in NaV1.7 availability may be underlying the effect of the toxins in both, C-LTMR fibers and C-HT nociceptors. Of course, non-specific actions of the toxins might produce unexpected but different effects in C-LTMR and C-HT, which could be based on their perhaps different NaV expression profile. It has to be considered also that NaV1.3 can generate ramp currents upon slow depolarising stimuli [29] This sub-type, however, is expressed mainly during foetal development or following nerve injury [30,31] and we therefore would not expect NaV1.3 to play a major role in C-HT or C-LTMRs responses. Alternatively, the presence of NaV1.6 along unmyelinated axons could contribute to axonal signal transmission [16], but its fast closed-state inactivation kinetics makes NaV1.6 unlikely to generate ramp currents responding to slow depolarising stimuli [32].

Protoxin did not reduce suprathreshold firing at higher stimulus intensities. It may be speculated that this observation is based on the slow recovery from fast inactivation of NaV1.7 [32,33]. This would oppose a major role of it in repetitive firing, but it was demonstrated recently that NaV1.7 indeed can contribute to repetitive firing, particularly when almost all TTX resistant NaV1.8 channels are unavailable [10]. Of note, NaV1.8 has fast repriming kinetics and thus facilitates high frequency discharge, for which it was expected that blockade of NaV1.7 or TTX has little influence on it [3]. Perhaps, only a small fraction of available NaV1.8 channels are needed to enable high firing rates and that are facilitated by NaV1.7 [10]. On the other hand, NaV1.6 fast activation and fast inactivation kinetics with rapid repriming times, tenfold faster than NaV1.7 and twofold faster than NaV1.3, are rather compatible with a role in burst firing [34]. It was also seen in human DRG patch-clamp recordings under concomitant NaV1.7 and NaV1.8 blockade that a prominent TTX-S current was still present in 2 of 8 recorded neurons [10]. Because the time of voltage-gated sodium channels to recover from inactivation is directly correlated with the capability of a neuron to repetitively discharge, fibers also expressing NaV1.6 channels can follow much higher frequencies upon stimulation compared to NaV1.7 alone [32] which might fit to the unexpectedly high discharge frequencies (>100 Hz) of C-LTMR [35].

It was observed that after TTX blockade, but not after lidocaine administration, C-fibers still responded in a synchronized fashion to sine 4 Hz stimulation, probably based on TTX-R channels, particularly NaV1.8 [36,37]. The more depolarised activation thresholds of NaV1.8 [38,39] could explain the increased delay of AP generation following TTX (Fig 4). Blocking NaV1.6 with µ-conotoxin have also increased initiation delay when compared to baseline at the same C-CAP current intensity and that provides indirect evidence that C-LTMRs dominates the rising phase of the C-CAP signals, once those fibers have the highest conduction velocity among C-fiber classes. There was also an increase in initiation delay after protoxin (S2A Fig) however non-significant. This more blurred effect may be linked to a different proportion of afferent and efferent nerve fiber classes in the tested fascicles. Another explanation derives from dynamic-clamp recordings suggesting that at depolarised states close to threshold NaV1.8 is the main contributor during the action potential [40], and thus residual TTX-resistant currents may contribute to preserved initiation delays in presence of protoxin. Such interactions might distort an expected clear-cut effect from the toxins. Moreover, in single fiber recordings, current spread must be considered when high stimulus intensities are applied after toxin administration. Higher current amplitudes could excite the nerve fiber at a more proximal site, skipping the site where the initial stimulus was delivered and potentially even skipping the zone in which the toxin was injected.

## Conclusions

We transferred a C-fiber specific electrical stimulation paradigm that has been successfully used for *in vivo* application in healthy humans and chronic itch/pain patients [4,5,11,41] to pig nerve fibers in which primary afferent fiber classes correspond remarkably well to humans [6]. While we could not confirm that protoxin and TTX delay the spike initiation upon slow depolarization, the toxins reduced the discharge induced by low-intensity phasic (1 Hz) sinusoidal stimulation in C-polymodal nociceptors but not C-LTMR fibers. In contrast, tonic firing for 1 minute at 4 Hz was reduced in both fiber classes indicating differential functional roles for NaV1.7 between C-nociceptors and low-threshold mechanosensitive C- fibers. Our results suggest that peripheral NaV1.7 may increase depolarization levels of C-nociceptors, and thus could impact clinically relevant ongoing pain.

## Supporting information

S1 Fig Concentration-response curves. TTX Concentration-response curves for SNF recordings were performed at 10nM, 100nM and 1µM concentrations. 1 Hz sinusoidal electrical stimulus induced APs at supra-maximum sine were collected and sigmoidal fitted. The concentration of 50% inhibition (IC50) in mean (SEM) for C-HT (S1A) was 0.58 (0.15) µM and for C-LTMR (S1B) was 0.83 (0.22) µM.
(TIF)

S2 Fig Latencies normalized to threshold stimulus intensity. (S2A) Increasing stimulus intensity reduces C-CAP time of initiation (top panel). By comparing time of initiation to baseline C-CAP at the same stimulus intensity before and after toxin administration, it was observed that both TTX and CTX but not protoxin showed a significant increase in sine wave C-CAP initiation delay (n = 13, Kruskal-Wallis, Dunn’s post hoc test, CTX p = 0.04, protoxin p = 0.059, TTX p = 0.0014, bottom panel). The effect of the toxins on C-HT and C-LTMR fibers was controlled by normalizing the time (1 Hz) and phase (4 Hz) of first AP generation to baseline and compared to equal stimulus intensities before and after the toxins were injected into the receptive field of the recorded unit. There was no effect of TTX or protoxin in either time (S2B, 1 Hz stimulus), (Kruskal-Wallis, p = 0.315) or phase (S2C, 4 Hz stimulus) (p = 0.414) of first AP generation in C-HT nociceptors. Similarly, there was no effect on C-LTMR fibers on time (S2D, Kruskal-Wallis, p = 0.089) or phase (S2E, p = 0.12) of first AP by either protoxin or TTX. Levels of significance are indicated as *p < 0.05 and **p < 0.01.
(TIF)

S1 Table Sine wave- number of action potentials. Mean ± SEM of number of action potentials for C-LTMR fibres and C-HT nociceptors tested with sine wave 1 Hz and 4 Hz at all intensity ranges before and after injection of Protoxin and TTX. Data was analyzed with Mixed design two-way ANOVA, Tukey post-hoc test. Levels of significance are indicated as *p < 0.05, **p < 0.01, ***p < 0.001 or ****p < 0.0001 (represented as #p < 0.05 for protoxin).
(DOCX)

S2 Table Sine wave- current and charge at threshold. Mean ± SEM of current at threshold (considered as a minimum response of 3 or more APs) for C-HT and C-LTMR nociceptors tested with sine wave 1 Hz. TTX increased the amount of current needed to initiate a response to 1 Hz stimulus in C-HT nociceptors (One-way ANOVA, Tukey post-hoc test, p = 0.0003), but not in C-LTMR fibers (p = 0.58). The charge at first AP for sine wave 4 Hz is shown for all intensity ranges before and after injection of Protoxin and TTX. There was no effect of both protoxin or TTX in increasing the charge at the first 4 Hz induced AP.
(DOCX)
