# Supplementary figures and images for: Assessing NaV1.7 during tonic firing in pig C-nociceptors
Source: PLoS One. 2025 Dec 3;20(12):e0335081. doi: 10.1371/journal.pone.0335081 (PMC12674544; doi:10.1371/journal.pone.0335081)

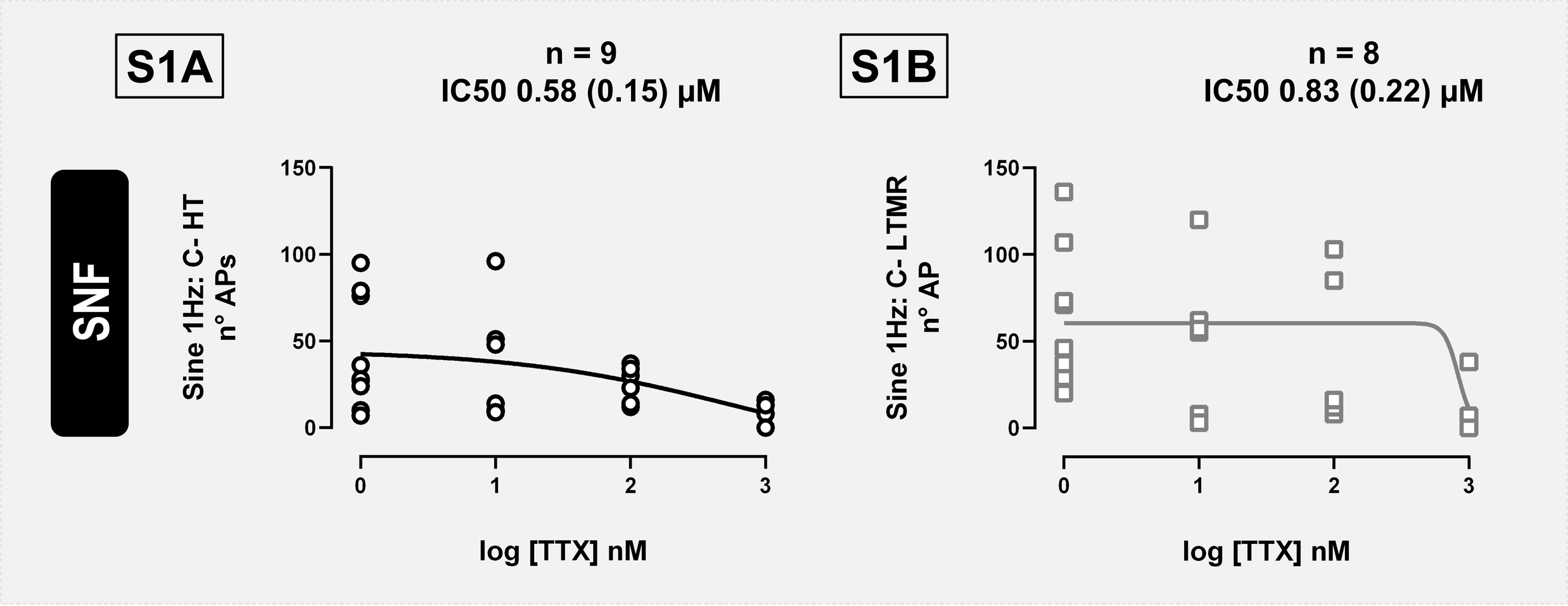

Supplement: S1 Fig — TTX Concentration-response curves for SNF recordings were performed at 10nM, 100nM and 1µM concentrations. 1 Hz sinusoidal electrical stimulus induced APs at supra-maximum sine were collected and sigmoidal fitted. The concentration of 50% inhibition (IC50) in mean (SEM) for C-HT (S1A) was 0.58 (0.15) µM and for C-LTMR (S1B) was 0.83 (0.22) µM. (TIF) [file pone.0335081.s001.tif]

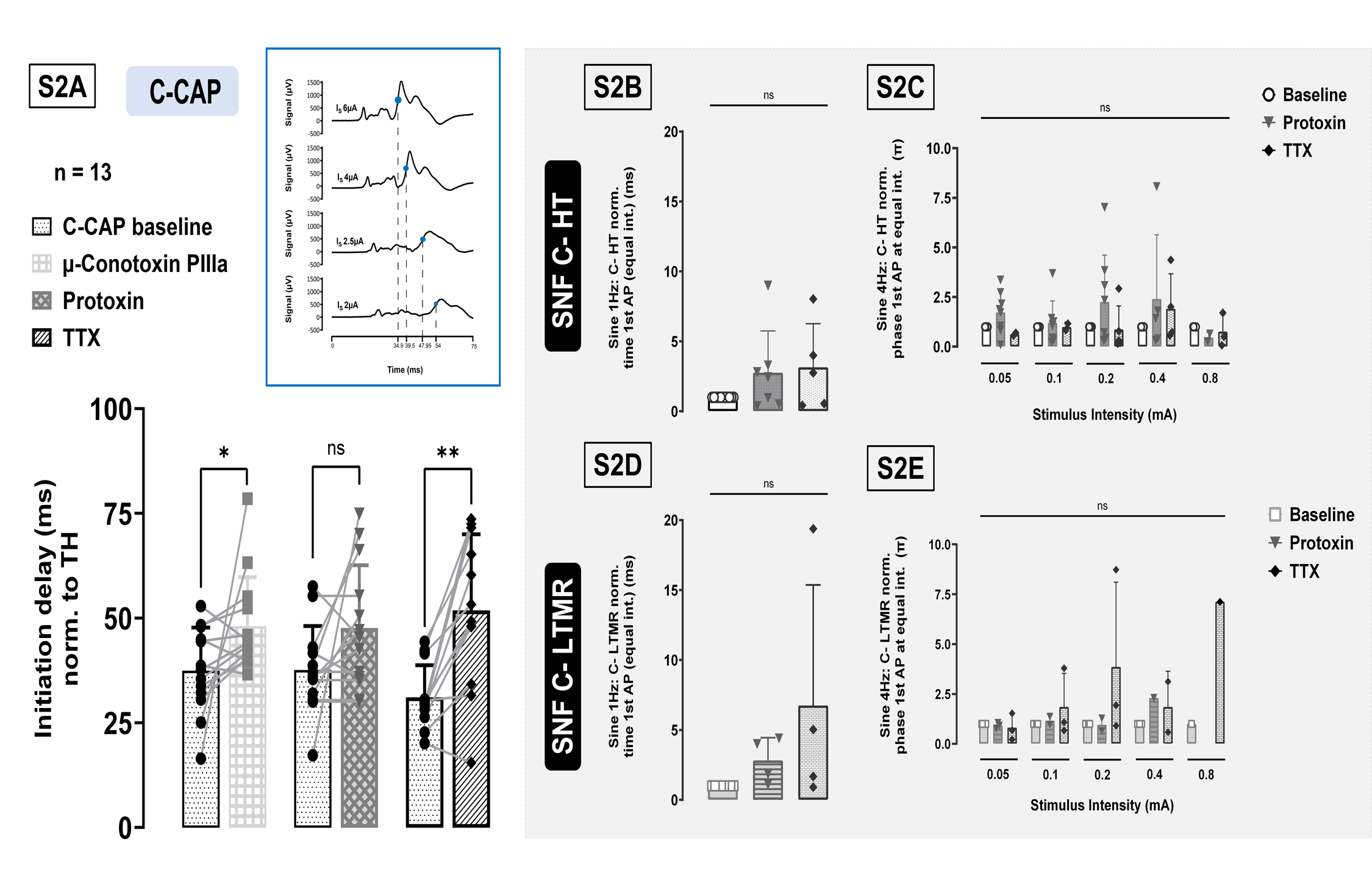

Supplement: S2 Fig — (S2A) Increasing stimulus intensity reduces C-CAP time of initiation (top panel). By comparing time of initiation to baseline C-CAP at the same stimulus intensity before and after toxin administration, it was observed that both TTX and CTX but not protoxin showed a significant increase in sine wave C-CAP initiation delay (n = 13, Kruskal-Wallis, Dunn’s post hoc test, CTX p = 0.04, protoxin p = 0.059, TTX p = 0.0014, bottom panel). The effect of the toxins on C-HT and C-LTMR fibers was controlled by normalizing the time (1 Hz) and phase (4 Hz) of first AP generation to baseline and compared to equal stimulus intensities before and after the toxins were injected into the receptive field of the recorded unit. There was no effect of TTX or protoxin in either time (S2B, 1 Hz stimulus), (Kruskal-Wallis, p = 0.315) or phase (S2C, 4 Hz stimulus) (p = 0.414) of first AP generation in C-HT nociceptors. Similarly, there was no effect on C-LTMR fibers on time (S2D, Kruskal-Wallis, p = 0.089) or phase (S2E, p = 0.12) of first AP by either protoxin or TTX. Levels of significance are indicated as *p < 0.05 and **p < 0.01. (TIF) [file pone.0335081.s002.tif]
