# Supplementary material for: Assessing NaV1.7 during tonic firing in pig C-nociceptors
Source: PLoS One. 2025 Dec 3;20(12):e0335081. doi: 10.1371/journal.pone.0335081 (PMC12674544; doi:10.1371/journal.pone.0335081)
Supplement: S1 Table — Mean ± SEM of number of action potentials for C-LTMR fibres and C-HT nociceptors tested with sine wave 1 Hz and 4 Hz at all intensity ranges before and after injection of Protoxin and TTX. Data was analyzed with Mixed design two-way ANOVA, Tukey post-hoc test. Levels of significance are indicated as *p < 0.05, **p < 0.01, ***p < 0.001 or ****p < 0.0001 (represented as #p < 0.05 for protoxin). (DOCX) [file pone.0335081.s003.docx]

| **Sine 1 Hz: Number of action potentials per pulse** | | | | | | |
| --- | --- | --- | --- | --- | --- | --- |
| C-LTMR | | | | | | |
| Baseline | | | Protoxin | | TTX 1µM | |
| Curr. (mA) | n | Mean ± SEM | n | Mean ± SEM | n | Mean ± SEM |
| 0.02 | 27 | 2.07 ± 0.64 | 5 | 0.20 ± 0.20 | 8 | 0.63 ± 0.32 |
| 0.04 | 28 | 3.39 ± 0.66 | 5 | 1.80 ± 0.97 | 8 | 1.25 ± 0.77 |
| 0.06 | 28 | 4.46 ± 0.88 | 5 | 2.60 ± 1.40 | 8 | 1.25 ± 0.84 |
| 0.08 | 28 | 4.86 ± 0.87 | 5 | 3.60 ± 2.29 | 8 | 1.75 ± 1.03 |
| 0.1 | 28 | 5.39 ± 0.90 | 5 | 3.60 ± 2.40 | 8 | 2.13 ± 1.37 |
| 0.2 | 27 | 7.22 ± 1.20 | 8 | 2.88 ± 1.75 | 10 | 2.40 ± 1.38 |
| 0.4 | 27 | 7.85 ± 1.23 | 8 | 3.88 ± 2.26 | 10 | 2.30 ± 1.20 |
| 0.6 | 27 | 8.04 ± 1.25 | 8 | 4.13 ± 2.05 | 10 | 2.70 ± 1.59 |
| 0.8 | 27 | 8.56 ± 1.20 | 8 | 4.50 ± 2.22 | 10 | 2.50 ± 1.47 |
| 1 | 27 | 8.33 ± 1.24 | 8 | 4.88 ± 2.49 | 10 | 2.30 ± 1.16 |
| 2 | 7 | 7 ± 2.92 | 3 | 6.67 ± 6.67 | 10 | 1.80 ± 1.59 |
| 4 | 7 | 8.14 ± 2.72 | 3 | 5.67 ± 5.67 | 10 | 2.70 ± 1.80 |
| 6 | 7 | 9.86 ± 2.91 | 3 | 6.33 ± 5.84 | 10 | 2.10 ± 1.30 |
| 8 | 7 | 9.57 ± 2.18 | 3 | 5 ± 4.51 | 10 | 2.80 ± 2.06 |
| 10 | 7 | 8.86 ± 2.18 | 3 | 4.67 ± 3.18 | 10 | 2.50 ± 1.46 |
| C-HT | | | | | | |
| Baseline | | | Protoxin | | TTX 1µM | |
| Curr. (mA) | n | Mean ± SEM | n | Mean ± SEM | n | Mean ± SEM |
| 0.02 | 36 | 0.69 ± 0.23 | 11 | 0.18 ± 0.18 | 23 | 0.26 ± 0.13 |
| 0.04 | 36 | 1.94 ± 0.51 | 11 | 0.27 ± 0.19 | 23 | 0.43 ± 0.29 |
| 0.06 | 36 | 2.78 ± 0.57 | 11 | 0.64 ± 0.39 | 23 | 0.48 ± 0.31* |
| 0.08 | 36 | 3.11 ± 0.52 | 11 | 0.73 ± 0.38 | 23 | 0.70 ± 0.38** |
| 0.1 | 36 | 3.28 ± 0.54 | 11 | 1.73 ± 0.82 | 23 | 0.78 ± 0.50* |
| 0.2 | 30 | 4.40 ± 0.65 | 11 | 2.64 ± 1.09 | 27 | 1.04 ± 0.55** |
| 0.4 | 30 | 6.47 ± 0.81 | 11 | 4.09 ± 1.36 | 27 | 1.22 ± 0.50**** |
| 0.6 | 30 | 7.20 ± 0.70 | 11 | 5.00 ± 1.47 | 27 | 1.48 ± 0.67**** |
| 0.8 | 30 | 6.97 ± 0.80 | 11 | 5.91 ± 1.66 | 27 | 1.93 ± 0.82** |
| 1 | 30 | 7.40 ± 0.73 | 11 | 6.55 ± 1.82 | 27 | 1.78 ± 0.72*** |
| 2 | 5 | 3.20 ± 1.02 | 5 | 4.00 ± 1.70 | 25 | 1.68 ± 0.75 |
| 4 | 5 | 6 ± 1.95 | 5 | 4.00 ± 1.41 | 25 | 2.48 ± 0.90 |
| 6 | 5 | 8 ± 2.07 | 5 | 3.40 ± 1.78 | 25 | 2.12 ± 0.78 |
| 8 | 5 | 6 ± 1.22 | 5 | 3.20 ± 1.69 | 25 | 2.44 ± 0.97 |
| 10 | 5 | 5.20 ± 1.85 | 5 | 3.40 ± 1.36 | 25 | 2.12 ± 0.68 |
| **Sine 4 Hz: Number of action potentials per pulse** | | | | | | |
| C-LTMR | | | | | | |
| Baseline | | | Protoxin | | TTX 1µM | |
| Curr. (mA) | n | Mean ± SEM | n | Mean ± SEM | n | Mean ± SEM |
| 0.05 | 28 | 0.16 ± 0.05 | 7 | 0.02 ± 0.01 | 12 | 0.03 ± 0.02 |
| 0.1 | 27 | 0.29 ± 0.08 | 7 | 0.01 ± 0.00 | 12 | 0.04 ± 0.02 |
| 0.2 | 25 | 0.50 ± 0.10 | 7 | 0.13 ± 0.10# | 12 | 0.12 ± 0.08** |
| 0.4 | 17 | 0.45 ± 0.10 | 5 | 0.03 ± 0.02# | 11 | 0.16 ± 0.10 |
| 0.8 | 10 | 0.61± 0.16 | 5 | 0.11 ± 0.11# | 9 | 0.13 ± 0.12** |
| 1.2 | 5 | 0.67 ± 0.30 | 4 | 0.00 ± 0.00# | 9 | 0.03 ± 0.03** |
| C-HT | | | | | | |
| Baseline | | | Protoxin | | TTX 1µM | |
| Curr. (mA) | n | Mean ± SEM | n | Mean ± SEM | n | Mean ± SEM |
| 0.05 | 29 | 0.16 ± 0.05 | 11 | 0.05 ± 0.02 | 14 | 0.01 ± 0.01 |
| 0.1 | 29 | 0.21 ± 0.06 | 11 | 0.11 ± 0.06 | 14 | 0.07 ± 0.04 |
| 0.2 | 27 | 0.25 ± 0.06 | 10 | 0.23 ± 0.09 | 14 | 0.08 ± 0.05* |
| 0.4 | 27 | 0.48 ± 0.08 | 8 | 0.23 ± 0.11## | 14 | 0.06 ± 0.04**** |
| 0.8 | 9 | 0.26 ± 0.20 | 6 | 0.19 ± 0.08# | 10 | 0.08 ± 0.06*** |
| 1.2 | 3 | 0.05 ± 0.14 | 4 | 0.06 ± 0.04 | 11 | 0.02 ± 0.01 |
