# Supplementary material for: Assessing NaV1.7 during tonic firing in pig C-nociceptors
Source: PLoS One. 2025 Dec 3;20(12):e0335081. doi: 10.1371/journal.pone.0335081 (PMC12674544; doi:10.1371/journal.pone.0335081)
Supplement: S2 Table — Mean ± SEM of current at threshold (considered as a minimum response of 3 or more APs) for C-HT and C-LTMR nociceptors tested with sine wave 1 Hz. TTX increased the amount of current needed to initiate a response to 1 Hz stimulus in C-HT nociceptors (One-way ANOVA, Tukey post-hoc test, p = 0.0003), but not in C-LTMR fibers (p = 0.58). The charge at first AP for sine wave 4 Hz is shown for all intensity ranges before and after injection of Protoxin and TTX. There was no effect of both protoxin or TTX in increasing the charge at the first 4 Hz induced AP. (DOCX) [file pone.0335081.s004.docx]

| **Sine 1 Hz: current at threshold (>=3 APs)** | | | | | | | | |
| --- | --- | --- | --- | --- | --- | --- | --- | --- |
| **C-HT** | | | | | | | | |
| n | Baseline | | | n | Protoxin | | n | TTX |
| 35 | 0.4 ± 0.17 | | | 10 | | 0.79 ± 0.37 | 9 | 2.49 ± 0.69*** |
| **C-LTMR** | | | | | | | | |
| 27 | 0.69 ± 0.27 | | | 5 | | 0.65 ± 0.36 | 5 | 2.86 ± 1.94 |
| **Sine 4 Hz: charge at first AP** | | | | | | | | |
| **C-HT** | | | | | | | | |
| Curr. (mA) | | n | Baseline | n | | Protoxin | n | TTX |
| 0.05 | 24 | | 0.05 ± 0.01 | 8 | | 0.05 ± 0.01 | 2 | 0.04 ± 0.03 |
| 0.1 | 24 | | 0.1 ± 0.01 | 9 | | 0.08 ± 0.02 | 4 | 0.14 ± 0.02 |
| 0.2 | 26 | | 0.17 ± 0.03 | 9 | | 0.15 ± 0.04 | 6 | 0.15 ± 0.06 |
| 0.4 | 25 | | 0.24 ± 0.04 | 5 | | 0.15 ± 0.03 | 6 | 0.28 ± 0.09 |
| 0.8 | 9 | | 0.81 ± 0.21 | 5 | | 0.29 ± 0.07 | 3 | 0.16 ± 0.09 |
| 1.2 | 3 | | 0.86 ± 0.16 | 2 | | 0.15 ± 0.01 | 3 | 0.94 ± 0.73 |
| **C-LTMR** | | | | | | | | |
| 0.05 | 20 | | 0.06 ± 0.01 | 3 | | 0.05 ± 0.01 | 4 | 0.03 ± 0.01 |
| 0.1 | 19 | | 0.09 ± 0.02 | 2 | | 0.08 ± 0.03 | 4 | 0.11 ± 0.03 |
| 0.2 | 17 | | 0.15 ± 0.04 | 3 | | 0.12 ± 0.07 | 5 | 0.20 ± 0.05 |
| 0.4 | 10 | | 0.23 ± 0.08 | 3 | | 0.29 ± 0.10 | 4 | 0.40 ± 0.11 |
| 0.8 | 5 | | 0.28 ± 0.12 | 2 | | 0.22 ± 0.15 | 5 | 0.71 ± 0.24 |
| 1.2 | 2 | | 0.34 ± 0.17 | 1 | | 0.81 | 3 | 0.88 ± 0.66 |
